# Complete Genome Sequences of Lytic Polysaccharide-Degrading Klebsiella pneumoniae Bacteriophages vB_KpnS_FZ10, vB_KpnP_FZ12, vB_KpnM_FZ14, and vB_KpnS_FZ41

**DOI:** 10.1128/MRA.00914-19

**Published:** 2019-09-26

**Authors:** Fedor Zurabov, Evgeniy Zhilenkov

**Affiliations:** a Research and Production Center, MicroMir, LLC, Moscow, Russia; DOE Joint Genome Institute

## Abstract

Members of the genus *Klebsiella* are among the leading microbial pathogens associated with nosocomial infection. At the same time, most nosocomial infections are caused by strains resistant to antibiotics. Here, we announce the complete genome sequences of four lytic polysaccharide-degrading bacteriophages, which will be used in complex therapeutic preparations.

## ANNOUNCEMENT

Klebsiella pneumoniae is a Gram-negative nonmotile encapsulated rod-shaped bacterium that is associated with nosocomial respiratory tract and urinary tract infections. Respiratory tract infections are among the most common infections in the world, and the clinical picture is rapidly progressing, as these infections lead to a high mortality rate (up to 50%). Most nosocomial infections are caused by strains resistant to antibiotics, which complicates their treatment (1). Lytic bacteriophages are natural agents that can help in their treatment.

*Klebsiella* phages vB_KpnS_FZ10, vB_KpnP_FZ12, vB_KpnM_FZ14, and vB_KpnS_FZ41 were isolated in 4 separate enrichments from sewage water collected from a wastewater treatment plant in the Moscow region, Russia. Sodium phosphate buffer (pH 7.0) was added to the sewage water sample to a final concentration of 0.05 M, and NaCl was added to a final concentration of 1 M. The mixture was incubated for 60 minutes at 37°C and 100 rpm. The supernatant was then differentially centrifuged, first for 20 minutes at 6,800 × *g*, and then supernatant was taken and centrifuged for 2 hours at 96,200 × *g*. Afterward, the supernatant was removed and the pellet dissolved in 1 ml of 0.1 M Tris-HCl buffer (pH 7.0). The resulting suspension was subsequently filtered through 1.2-μm, 0.45-μm, and 0.22-μm filters. The filtrate was then incubated at 37°C for 24 hours in a flask with brain heart infusion (BHI) broth and one specific strain of Klebsiella pneumoniae for each phage (names and GenBank accession identification numbers [IDs] are indicated in Table 1) and then purified with differential centrifugation and filtration, as described above. After a spot test against the strain used for enrichment, the phage purification process was performed using Gratia titration (2), as follows: 0.1 ml of each serial dilution (10^−1^ to 10^−10^) of bacteriophage filtrate in physiological salt solution was mixed with 0.2 ml of Klebsiella pneumoniae suspension (10^9^ CFU) and 3 ml of soft agar and then added to a petri dish with bottom agar and incubated for 24 hours at 37°C. Afterward, a single plaque was picked, and the enrichment process was repeated (incubation with Klebsiella pneumoniae, differential centrifugation, and filtration). Finally, bacteriophage filtrate was purified using CsCl centrifugation.

**TABLE 1:** Genome data of bacteriophages vB_KpnS_FZ10, vB_KpnP_FZ12, vB_KpnM_FZ14, and vB_KpnS_FZ41

Bacteriophage	Strain NCBI ID	Genome size (bp)	G+C content (%)	Coverage (×)	No. of ORFs	Taxonomic identification (family, genus)	Related virus(es) (GenBank accession no.)	Identity (%) (query coverage [%])
vB_KpnS_FZ10	K. pneumoniae RV_BA_03_B LBK, 573	50,381	50.66	65.38	42	*Tunavirinae*, *Webervirus*	*Klebsiella* phage NJR15 (MH633487)	96.71 (94)
vB_KpnP_FZ12	K. pneumoniae subsp. *pneumoniae* DSM 30104^T^ HAM, 72407	39,519	53.06	71.03	43	*Podoviridae*, *Autographivirinae* (subfamily), *Przondovirus*	*Klebsiella* phage vB_KpnP_KpV763 (KX591654)	94.43 (93)
vB_KpnM_FZ14	K. pneumoniae subsp. *pneumoniae* 9295_1 CHB, 72407	49,370	48.58	71.60	35	*Myoviridae*, *Jedunavirus*	*Klebsiella* phage vB_KpnM_KpV52 (KX237516)	96.58 (79)
vB_KpnS_FZ41	K. pneumoniae subsp. *pneumoniae* DSM 30104^T^ HAM, 72407	106,104	45.22	72.25	103	*Siphoviridae*, *Sugarlandviru*s	*Klebsiella* phages vB_Kpn_IME260 (KX845404) and Sugarland (NC_042093)	96.77 (93), 97.52 (89)

Phage DNA was extracted using chloroform extraction (3). The DNA library was constructed using a Nextera DNA library preparation kit (Illumina, San Diego, CA) and sequenced with an Illumina HiSeq T1500 sequencer, resulting in approximately 1 million 2 × 250-bp paired-end reads. The quality control and primary processing were performed using FastQC and Trimmomatic (HEADCROP:20, SLIDINGWINDOW:3:24, MINLEN:200, CROP:200) (4). Coverage was normalized to 50× using BBNorm (https://jgi.doe.gov/data-and-tools/bbtools/). *De novo* assembly was performed using the MIRA assembler 4.9.6 (5). Contig completion was confirmed by comparison to closely related genomes known to be complete (GenBank accession numbers are presented in Table 1). An open reading frame search was performed using MetaProdigal 2.6 (6). Annotation was performed using peer-reviewed phage and bacterial proteins from UniProt (https://www.uniprot.org) and proteins from the following databases of determinants of antimicrobial resistance and bacterial virulence factors: Virulence Factor Database (VFDB) (7), Comprehensive Antibiotic Resistance Database (CARD) (8), Antibiotic Resistance Gene-ANNOTation (ARG-ANNOT) (9), and ResFinder (10). The remaining open reading frames (ORFs) were annotated with the hmmscan application (maximum E value, 0.001) (11) using all bacterial hidden Markov model (HMM) profiles from the Pfam database (https://pfam.xfam.org). The number of tRNAs was predicted using tRNAscan-SE 2.0 (12). All analyses except those indicated were performed using default parameters.

All phages have a double-stranded DNA. None of the ORFs were found to contain lysogenic, virulence, or antibiotic resistance sequences. Phages vB_KpnS_FZ10, vB_KpnP_FZ12, and vB_KpnM_FZ14 carry proteins with depolymerase activity, which may play a crucial role in Klebsiella pneumoniae capsule degradation (13). tRNAs were predicted only for vB_KpnS_FZ41 (25 tRNA ORFs). Details on genome size, content, coverage, number of ORFs, taxonomic identification, and related viruses are presented in Table 1. Average nucleotide identity and query coverage were calculated using BLASTn (https://blast.ncbi.nlm.nih.gov/Blast.cgi).

### Data availability.

The complete genome sequences of Klebsiella pneumoniae phages vB_KpnS_FZ10, vB_KpnP_FZ12, vB_KpnM_FZ14, and vB_KpnS_FZ41 have been deposited in GenBank under the accession numbers MK521904, MK521905, MK521906, and MK521907, respectively. Raw Illumina reads are available in the NCBI SRA under the accession numbers SRR10037530, SRR10037529, SRR10037528, and SRR10037527, respectively. The associated BioProject number is PRJNA562287.
